# Adherence to Mediterranean Diet: Any Association with NAFLD?

**DOI:** 10.3390/antiox12071318

**Published:** 2023-06-21

**Authors:** Luigi Barrea, Ludovica Verde, Silvia Savastano, Annamaria Colao, Giovanna Muscogiuri

**Affiliations:** 1 Dipartimento di Scienze Umanistiche, Università Telematica Pegaso, Centro Direzionale Isola F2, Via Porzio, 80143 Naples, Italy; 2 Centro Italiano per la cura e il Benessere del Paziente con Obesità (C.I.B.O), Unità di Endocrinologia, Diabetologia e Andrologia, Dipartimento di Medicina Clinica e Chirurgia, Università degli Studi di Napoli Federico II, Via Sergio Pansini 5, 80131 Naples, Italy; 3 Department of Public Health, University of Naples Federico II, Via Sergio Pansini 5, 80131 Naples, Italy; 4 Unità di Endocrinologia, Diabetologia e Andrologia, Dipartimento di Medicina Clinica e Chirurgia, Università degli Studi di Napoli Federico II, Via Sergio Pansini 5, 80131 Naples, Italy; 5 Cattedra Unesco “Educazione Alla Salute E Allo Sviluppo Sostenibile”, University Federico II, 80131 Naples, Italy

**Keywords:** Mediterranean diet, non-alcoholic liver disease, obesity, diet, nutrition, VAI, FLI, HoMA-IR

## Abstract

Oxidative stress is considered one of the main determinants in the pathophysiology of non-alcoholic fatty liver disease (NAFLD) and obesity. The alterations of oxidant/antioxidant balance are related to chronic impairment of metabolism leading to mitochondrial dysfunction. Increased oxidative stress also triggers hepatocytes stress pathways, leading to inflammation and contributing to the progression of non-alcoholic steatohepatitis (NASH). Currently, the first-line therapeutic treatment of NAFLD is based on lifestyle interventions, suggesting the Mediterranean Diet (MD) as a preferable nutritional approach due to its antioxidant properties. However, it is still debated if adherence to MD could have a role in determining the risk of developing NAFLD directly or indirectly through its effect on weight. We enrolled 336 subjects (aged 35.87 ± 10.37 years; BMI 31.18 ± 9.66 kg/m^2^) assessing anthropometric parameters, lifestyle habits, metabolic parameters (fasting plasma glucose, fasting plasma insulin, triglycerides (TG), total cholesterol, low-density (LDL) and high-density lipoprotein (HDL) cholesterol, alanine transaminase (ALT), aspartate aminotransferase (AST), and γ-glutamyltransferase (γGT), cardio-metabolic indices [Homeostatic Model Assessment Insulin Resistance (HoMA-IR), visceral adipose index (VAI) and fatty liver index (FLI)] and adherence to MD [with the PREvención con DIetaMEDiterránea (PREDIMED) questionnaire]. Subjects with NAFLD had significantly higher anthropometric parameters, cardio-metabolic indices and lower adherence to MD than subjects without NAFLD. In a multiple regression analysis, PREDIMED score was the main predictor of FLI (*p* < 0.001) and came in first, followed by HoMA-IR, while VAI was not a predictor. A PREDIMED score value of <6 could serve as a threshold to identify patients who are more likely to have NAFLD (*p* < 0.001). In conclusion, high adherence to MD resulted in a lower risk of having NAFLD. Adherence to MD could have a direct role on the risk of developing NAFLD, regardless of visceral adipose tissue.

## 1. Introduction

The prevalence of non-alcoholic fatty liver disease (NAFLD) has increased from 25% (1990–2006) to 38% (2016–2019), becoming a growing epidemic condition [1,2]. NAFLD encompasses the entire spectrum of fatty liver disease, ranging from simple steatosis to non-alcoholic steatohepatitis (NASH), characterized by inflammation with hepatocyte lesions and with or without varying degrees of fibrosis, to cirrhosis and hepatocellular carcinoma (HCC) [3,4]. 

NAFLD is considered a multifactorial disease in which environmental factors, such as nutrient intake and exposure, physical activity, genetics, epigenetics, and gut microbiota composition have been shown to interact with each other contributing to its development [5,6]. In addition, oxidative stress represents an important key mediator in low-grade inflammation in metabolic syndrome and mostly in the progression of NAFLD into NASH. 

Lifestyle management, such as diet and physical activity, aiming at controlling body weight and cardio-metabolic risk factors related to metabolic syndrome, is the basis of NAFLD treatment [7]. In this regard, the EASL-EASD-EASO clinical Practice Guidelines recommend as current treatment for NAFLD the Mediterranean diet (MD), an antioxidant nutritional approach rich in compounds, such as polyphenols, carotenoids, fiber, polyunsaturated fatty acids, low-refined and low-sugar foods, together with constant physical activity [4]. These recommendations are based on evidence that highlight the association between adherence to MD and NAFLD. Kontogianni et al. carried out a study on 73 subjects with obesity or overweight, of which 34 underwent liver biopsies [8]. Interestingly, a higher adherence to MD (as determined by the MedDiet Score) was associated with a lower likelihood to develop NAFLD [8]. This finding was also confirmed by Aller et al. who found subjects that more highly adhered to MD (as determined by the 14-item MD assessment tool) were less likely to develop NAFLD with severe histological features, as well as having insulin resistance [9]. Very recently, Baratta et al. found that adherence to MD was inversely associated with NAFLD prevalence (as evaluated by ultrasound) in a population of subjects with obesity or overweight at high risk of cardio-metabolic diseases [10]. In addition, a high adherence to MD was related to an increased chance to improve cardio-metabolic risk factors. However, low adherence to MD has also been detected in subjects with obesity [11]. 

Thus, the aim of our study was to investigate the association between adherence to MD and the development of NAFLD, while also considering the potential impact of visceral adiposity and insulin resistance on this association. By exploring the interplay between MD, visceral adiposity, and insulin resistance, our study aimed to provide a deeper understanding of the complex mechanisms underlying NAFLD development and potentially identify new avenues for prevention and treatment strategies.

## 2. Materials and Methods

This was a cross-sectional observational study carried out at the Department of Clinical Medicine and Surgery, Unit of Endocrinology, University Federico II, Naples (Italy). The study was carried out in accordance with the Code of Ethics of the World Medical Association (Declaration of Helsinki) for experiments involving humans, and was approved by the Ethical Committee of the University of Naples “Federico II” Medical School (n. 05/14). The purpose of the protocol was explained to all the study participants, and written informed consent was obtained.

### 2.1. Population Study

Recruitment strategies included a sample of 336 subjects (age 35.87 ± 10.37 years; BMI 31.18 ± 9.66 kg/m^2^) of both genders consecutively enrolled among patients of the outpatient clinic at the Department of Clinical Medicine and Surgery, Unit of Endocrinology, University Federico II, Naples (Italy). The recruitment began in January 2022 and ended in January 2023. A full medical history, including drug use, was collected. To increase the homogeneity of the subject sample, we included only adults of both genders with the following criteria of exclusion:

- Impaired renal function (estimated glomerular filtration rate ≥ 90 mL/min/1.73 m^2^ calculated according to Chronic Kidney Disease Epidemiology Collaboration (CKD-EPI) [12];
- Chronic liver diseases, viral hepatitis patients, hemochromatosis, hepatic malignancy;
- Presence of type 2 diabetes (T2DM) (according to the criteria of the American Diabetes Association (ADA) as follows: basal blood glucose ≥ 126 mg/dL on two occasions, or glycated haemoglobin (HbA1c) ≥ 6.5% (≥48 mmoL/moL) on two occasions, or both at the same time) [13]. Furthermore, participants on antidiabetic medication were considered to have T2DM;
- Clinical atherosclerosis (coronary artery disease, peripheral vascular disease);
- User of antibiotics or probiotics within two months of recruitment;
- Specific nutritional regimens, including vegan or vegetarian diets;
- Vitamin/mineral or antioxidant supplementation;
- Alcohol abuse according to the Diagnostic and Statistical Manual of Mental Disorders (DSM) V diagnostic criteria [14].

### 2.2. Anthropometric Measurements

Anthropometric measurements were carried out in the morning between 8 and 10 am, after the participants fasted overnight. A trained nutritionist carried out the measurements using standardized procedures. During the visit, the participants were asked to wear light clothes and no shoes. The height and weight of each participant were measured to calculate their body mass index (BMI) (weight in kg divided by height squared in m^2^, kg/m^2^). Height was measured with a wall-mounted stadiometer (Seca 711; Seca, Hamburg, Germany) to the nearest 0.5 cm, while body weight was determined using a calibrated balance beam scale (Seca 711; Seca, Hamburg, Germany) to the nearest 0.1 kg. BMI was classified according to the World Health Organization (WHO)’s criteria, which defines a normal weight as 18.5–24.9 kg/m^2^, overweight as 25.0–29.9 kg/m^2^, grade I obesity as 30.0–34.9 kg/m^2^, grade II obesity as 35.0–39.9 kg/m^2^, and grade III obesity as ≥40.0 kg/m^2^. The waist circumference (WC) was measured to the nearest 0.1 cm using a non-stretchable measuring tape at the natural indentation or at a midway level between the lower edge of the rib cage and iliac crest, if no natural indentation was visible [15]. In accordance with NCEP ATP III criteria we considered WC cut-offs indicative of abdominal obesity as 102 cm for men and 88 cm for women [16].

### 2.3. Adherence to Mediterranean Diet

Adherence to MD was assessed using the 14-item Prevención con Dieta Mediterránea (PREDIMED) questionnaire, which has been validated before [17]. As previously reported, a trained nutritionist administered the questionnaire to all study participants during an in-person interview [18,19]. Each item was assigned either a score of 1 or 0, and the PREDIMED score was calculated based on the total scores. A score of 0–5 indicated the lowest adherence to MD, a score of 6–9 indicated average adherence, and a score of ≥10 indicated the highest adherence to MD.

### 2.4. Physical Activity and Smoking Habits

Physical activity levels were assessed using a standardized questionnaire that asked participants whether they engaged in at least 30 minutes of aerobic exercise per day on a regular basis (yes or no responses). Similarly, smoking habits were also evaluated using a standard questionnaire (yes or no responses). Participants who had quit smoking at least one year prior to the interview were classified as “former smokers”, while those who smoked at least one cigarette per day were classified as “current smokers”. Participants who did not currently smoke and had not smoked in the past year were considered “non-current smokers”. For the purposes of the analysis, former and non-current smokers were grouped together as “non-smokers”. These methods have been used in previous studies [20,21,22,23].

### 2.5. Assay Methods

Blood specimens were collected in the morning between 8 and 10 am after the participants fasted for at least 8 h and were stored at a temperature of −80 °C until they were processed. All biochemical analyses, including fasting plasma glucose, total cholesterol, fasting plasma triglycerides (TG), alanine transaminase (ALT), aspartate aminotransferase (AST), and γ-glutamyltransferase (γGT), were conducted using a Roche Modular Analytics System in the Central Biochemistry Laboratory of the institution. The levels of low-density lipoprotein (LDL) cholesterol and high-density lipoprotein (HDL) cholesterol were determined using a direct method that uses a homogeneous enzymatic assay for the quantitative determination of LDL and HDL cholesterol. Fasting plasma insulin levels were measured using commercially available kits and a solid-phase chemiluminescent enzyme immunoassay. The intra-assay coefficients of variation were less than 5.5%, which has been previously reported [20,21,22,23].

### 2.6. Non-Alcoholic Fatty Liver Disease

The presence of NAFLD was assessed by means of the ‘fatty liver index’ (FLI). FLI was calculated using a specific validated formula and according to Bedogni’s criterion, an FLI ≥ 60 was considered as the cut-off value for indicating the presence of NAFLD [24].

FLI = eL/(1 + eL) × 100, L = 0.953 × loge TG + 0.139 BMI + 0.718 × logeγGT + 0.053 × WC − 15.745

FLI is an accurate and easy-to-use index, as BMI, WC, TG and γGT are routine measures in clinical practice.

### 2.7. Cardio-Metabolic Indices

Visceral adipose index (VAI) score was calculated separately for males and females using a sex-specific formula, and age-specific VAI cut-off values were applied based on Amato et al.’s research [25,26].

For males, the VAI score was calculated using the formula:

VAI = [WC/39.68 + (1.88 × BMI)] × (TG/1.03) × (1.31/HDL)

For females, the VAI score was calculated using the formula:

VAI = [WC/36.58 + (1.89 × BMI)] × (TG/0.81) × (1.52/HDL)

Homeostatic Model Assessment Insulin Resistance (HoMA IR) was calculated according to Matthews et al. and a value of HoMA-IR > 2.5 was used as cut-off of insulin resistance [27].

HoMA-IR = (fasting plasma glucose × fasting plasma insulin)/405

### 2.8. Power Size Justification

The power of the sample was calculated by the difference of means ± standard deviation (SD) of adherence to MD between subjects with FLI < 60 and subjects with FLI ≥ 60 (9.97 ± 2.33 vs. 6.01 ± 2.44, respectively). The number of cases required was 18 individuals for the two groups.

The calculated power size was 95%, with a type I (alpha) error of 0.05 (95%), and a type II (beta) of 0.05. The calculations of sample size and power were performed while using a sample size calculator Clinical Calc (https://clincalc.com/stats/samplesize.aspx, accessed on 1 January 2022), as previously reported [20,21,22,23].

### 2.9. Statistical Analysis

Continuous variables were expressed as mean ± SD whereas categorical variables were reported as numbers (n) and percentage (%). Kolmogorov–Smirnov test was used to test data distribution. 

Differences in age, anthropometric measurements, metabolic parameters, and cardio-metabolic indices in PREDIMED categories were analyzed by ANOVA test, with the Bonferroni test as a post-hoc test. Differences between FLI cutoff (<60 and ≥60) were analyzed by Student’s independent *t*-test. Correlations between study variables were performed using Pearson’s *r* correlation coefficients (continuous variables). A partial correlation analyses was used to determine the relationship between FLI and age, anthropometric measurements, metabolic parameters, and cardio-metabolic indices controlling for HoMA-IR and VAI. A multiple linear regression analysis models (stepwise method), expressed as R^2^, Beta (*β*), and *t*, with PREDIMED score as dependent variables were used to estimate the predictive value of cardio-metabolic indices. Receiver operator characteristic (ROC) curve analysis was performed to determine sensitivity and specificity, area under the curve (AUC), as well as cut-off values of PREDIMED score in detecting NAFLD. A *p* value < 0.05 was considered significant. Statistical analysis was performed according to standard methods using the Statistical Package for Social Sciences software 26.0 (SPSS/PC; SPSS, Chicago, IL, USA).

## 3. Results

Three hundred and thirty-six subjects (37.5% males and 62.5% females; aged 35.87 ± 10.37 years) were enrolled. In Table 1, sex, age, lifestyle habits, anthropometric measurements, metabolic parameters, and cardio-metabolic indices are reported. Normal weight was present in most (43.2%) of the enrolled subjects. Overweight was detected in 145 (13.4%) subjects of cohort, while grade I obesity was found in 38 subjects (11.3%), grade II obesity in 40 subjects (11.9%) and grade III obesity in 68 individuals (20.2%). The mean FLI was 53.77 ± 5.43. One hundred eighty-three (54.5%) subjects had FLI < 60, while 153 (45.6%) subjects had FLI ≥ 60.

In Table 2, response frequency of dietary components included in PREDIMED questionnaire and adherence to MD of the entire study population are reported. Extra virgin olive oil was the most consumed food item (83.3%), followed by red processed meats with poultry in third position. Seventy-six subjects (22.6%) reported low adherence to MD, 152 subjects (45.2%) reported average adherence to MD, while 108 subjects (32.2%) reported high adherence to MD.

Study participants’ characteristics grouped according to the degree of adherence to MD are summarized in Table 3. As shown, subjects with low adherence to MD presented significant higher values of BMI and WC than subjects with average and high adherence to MD. In addition, subjects with low adherence to MD had significantly higher fasting plasma glucose, fasting plasma insulin, LDL cholesterol, TG, AST ALT, and γGT than subjects with average and high adherence to MD. In addition, cardio-metabolic indices such as HoMA-IR, VAI and FLI were significantly higher in subjects with low adherence to MD than subjects with average and high adherence to MD. HDL cholesterol was significantly lower in subjects with low adherence to MD compared to the other two groups. Interestingly, subjects with average adherence to MD had significantly higher BMI and WC than subjects with high adherence to MD. In the same manner, they had significantly higher fasting plasma glucose, fasting plasma insulin, LDL cholesterol, TG, AST, ALT, γGT than subjects with high adherence to MD. No differences were observed in age and male-to-female ratio. Cardio-metabolic indices, such as HoMA-IR, VAI and FLI, were significantly higher in subjects with average adherence to MD than subjects with high adherence to MD. HDL cholesterol was significantly lower in subjects with average adherence to MD compared to subjects with high adherence to MD. No differences were detected in terms of age among the three groups.

Subjects with NAFLD (FLI ≥ 60) had significantly higher BMI and WC values compared to subjects without NAFLD (FLI < 60) (Table 4). Metabolic parameters such as fasting plasma glucose, fasting plasma insulin, LDL cholesterol, HDL cholesterol, TG, AST, ALT, γGT were significantly higher in subjects with NAFLD than subjects without NAFLD. Cardio-metabolic indices such as HoMA-IR, VAI and FLI were significantly higher in subjects with NAFLD than subjects without NAFLD. In addition, subjects with NAFLD (FLI ≥ 60) had significantly lower PREDIMED scores and a higher percentage of subjects with average adherence to the MD. No differences between the two groups were detected regarding age.

The correlations of FLI with anthropometric measurements, metabolic parameters, cardio-metabolic indices and PREDIMED score are summarized in Table 5. FLI showed significant positive correlations with all anthropometric measurements, metabolic parameters and cardio-metabolic indices. Significant negative correlations of FLI with HDL cholesterol and PREDIMED score were observed. These correlations remained significant even after adjustment for VAI and HOMA-IR.

To compare the relative predictive power of the cardio-metabolic indices associated with PREDIMED score, we performed a multiple linear regression analysis using a model that included as HoMA-IR, VAI, and FLI (Table 6). Using this model, FLI entered at the first step (*p* < 0.001), while HoMA-IR and VAI were excluded. To compare the relative predictive power of FLI and the cardio-metabolic indices associated with PREDIMED score, we performed a second multiple linear regression analysis model. Using this second model, FLI entered at the first step (*p* < 0.001), while VAI were excluded.

**Figure 1:**
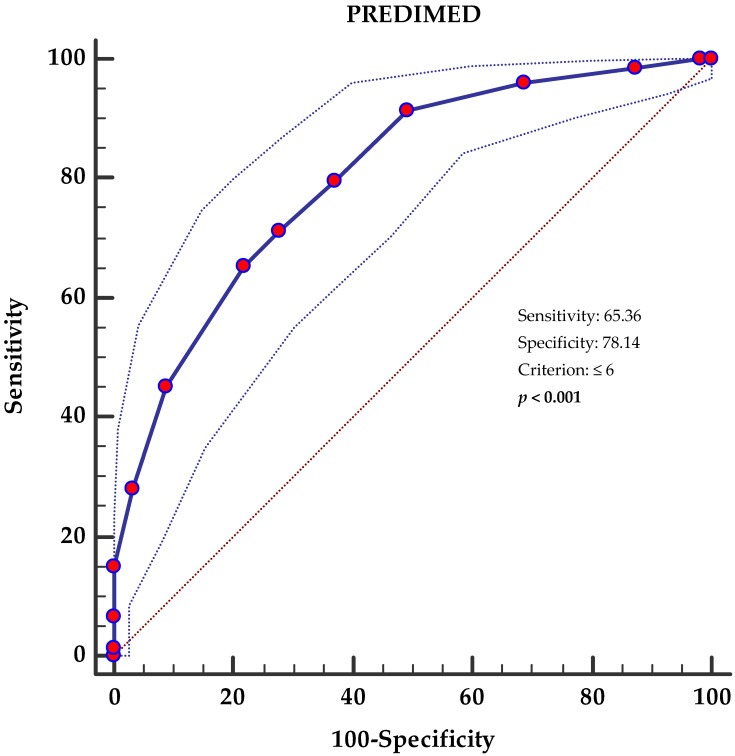
Receiver operator characteristic (ROC) for NAFLD’s (FLI ≥ 60) predictive values of PREDIMED score. The 45° diagonal line serves as a reference line, as it is the ROC curve of the random classification. The line with the red dots represents the distribution of the data while the two dashed lines outside it represent the confidence intervals. The point furthest northwest of the ROC curve corresponds to the best cut-off, in the sense of maximizing the number of correctly classified subjects (and minimizing the number of subjects with a false diagnosis). A *p*-value in bold denotes a significant difference (*p* < 0.05). PREDIMED, PREvención con DIetaMEDiterránea.

An ROC curve analysis was then performed to identify the cut off value of the PREDIMED score that was predictive of NAFLD (FLI ≥ 60). Specifically, PREDIMED scores < 6 (*p* < 0.001, AUC 0.804, standard error 0.0235, 95% CI 0.75 to 0.85 (Figure 1)) were identified as the thresholds for NAFLD (FLI ≥ 60).

## 4. Discussion

In this study, we demonstrated that both MD and insulin resistance might impact the risk of NAFLD. Specifically, we showed that increased HoMA-IR, together with low adherence to MD, were independently and significantly associated with NAFLD, while visceral adipose tissue calculated as VAI was not. Interestingly in our cohort, subjects with NAFLD showed higher anthropometric measurements, metabolic parameters and cardio-metabolic indices than subjects without NAFLD. Subjects with NAFLD had significantly lower values of PREDIMED score, i.e., lower adherence to MD than subjects without NAFLD. 

The link between NAFLD and MD has been previously reported in 243 youths with overweight/obesity with and without NAFLD in which adherence to MD was assessed by the KIDMED score [28]. The key finding of this study was the existence of the negative association between the extent of liver damage and the level of adherence to MD, indicating that adherence to MD may play a role in the severity of liver damage in relation to NALFD. 

Further, we identified a cut off of PREDIMED score < 6 as a tool to be used to screen subjects at high risk of having NAFLD. Our results are therefore consistent, but also bring up and reinforce the concept that adherence of MD independently of visceral adipose tissue influences NAFLD, as demonstrated by the results of our regression analysis. In agreement with our results, Trovato et al. carried out a study in 1199 subjects with overweight/obesity with and without ultrasound-diagnosed hepatic steatosis, finding that patients with NAFLD were less adherent to MD, but interestingly adherence to MD predicted the occurrence of NAFLD independently of BMI [29]. In addition, the same authors evaluated the effect of MD intervention on the Bright Liver Score at baseline and after 1, 3 and 6 months [30]. Over a 6-month period the reduction of fat liver content was greater the higher the adherence to MD, and interestingly, the effect of MD was independent of other lifestyle changes [30]. These finding are also reported by other similar studies [31,32].

The direct effect of MD on NAFLD, both on pathogenesis and on risk of progression from NAFLD to NASH, could be explained by antinflammatory and antioxidant properties of MD [33,34]. Indeed, MD contains compounds such as polyphenols, vitamins and other molecules that play a role in this sense. Polyphenols are in whole-grain cereals, vegetables and fresh fruits, red wine, olive oil and nuts. They have a phenolic structure, and they are a heterogenic group consisting of flavonoid polyphenols and non-flavonoid polyphenols based on their chemical structure [34]. Flavonoids exert a hepatoprotective effect due to their antioxidant and anti-inflammatory properties [34,35]. In addition, resveratrol, a non-flavonoid mostly present in red wine, has been reported to interact with homeostasis of vessels, blood platelets and the clotting and fibrinolytic system of plasma, thus exerting hepato-protective properties [36,37]. Vitamins, such as vitamin E, D, and C, are components of MD and they have been shown to play antioxidant properties, too [38,39,40]. In addition, lycopene, a carotenoid found in several fruits and vegetables of the MD, also has been demonstrated to exert a preventive effect on experimental NASH through the reduction of steatosis, inflammation, and oxidative stress in animal studies [41]. High MUFA content along with a balanced PUFA omega 6-to-omega 3 ratio is another key player of MD in preventing the development of NAFLD by improving plasma lipid levels, decreasing body fat and reducing postprandial adiponectin expression [42,43]. 

The liver is closely related to the gut since it receives about 70% of its blood supply from the intestine though the portal vein [44,45]. The tight relationship between NAFLD and gut microbiota has been highlighted by several studies [44,45]. In this context, MD could also have a beneficial role on NAFLD acting through gut microbiota. Indeed, high dietary fiber intake contained in MD reduces *Firmicutes* and increased *Bacteroidetes*, thus promoting a microbial pattern that improves obesity, inflammation, and related metabolic alterations [46]. Moreover, polyphenols promote the increase of *Bifidobacterium*, that are well known for their properties in reducing plasma cholesterol and C-reactive protein [46]. Interestingly, we found an independent association of HoMA-IR with NAFLD. Insulin resistance is a well-established risk factor for the onset of NAFLD and its progression to NASH [47]. Indeed, in the insulin resistance state, there is an impaired uptake synthesis, export, and oxidation of fatty free acids that results in fat accumulation. Interestingly, this mechanism is independent of obesity, as we found, and as has been demonstrated in subjects without obesity [48].

Moreover, in the insulin resistant condition, the liver does not suppress hepatic glucose production in response to insulin and increases de novo lipogenesis, activating the Notch signaling pathway; [49] in fact, in subjects with NAFLD the increase in de novo lipogenesis is 5-fold higher than in healthy subjects [49]. 

One of the main limitations of our study was the cross-sectional design that provides information on associations of parameters, but not on causality. In addition, although the sample size could seem relatively small, it was calculated by using 95% statistical power to detect statistical significance of the results with adequate power. The study population from an outpatient clinic was another limitation of the study. However, it is important to study patients from an outpatient clinic, as studying this specific population can provide valuable insights into clinical aspects, disease management and treatment outcomes in a healthcare setting. However, further population-based or multicenter trial studies remain necessary to validate the results in more diverse populations, including individuals outside the outpatient setting. Finally, although we observed a significant association between adherence to MD and NAFLD, it is important to recognize the possibility of residual confounding of potential unmeasured variables in the study. However, to mitigate the impact of residual confounding, we used appropriate statistical techniques, such as multivariable regression models, to adjust for known confounders. To overcome this limitation, future studies might consider including a more complete set of confounding variables in their analyses.

The strength of our study lies on the accurate characterization of enrolled subjects by a trained team of Endocrinologists and Nutritionists. To ensure the homogeneity of the sample, all enrolled subjects came from the same geographical area with similar nutritional habits. Third, our study highlights the role of MD in the onset of NAFLD and it was the first to identify a cutoff of PREDIMED score as an easy tool to screen subjects at risk of having NAFLD.

## 5. Conclusions

In conclusion, this study shows that low adherence to MD and insulin resistance were associated with the risk of developing NAFLD. We found compelling evidence indicating that NAFLD was independently and significantly associated with both increased HoMA-IR and low adherence to MD. Notably, our findings revealed that the calculated measure of visceral adipose tissue known as VAI did not demonstrate a main association with NAFLD. Therefore, it might provide the rational basis for a personalized management of patients with NAFLD taking into account nutritional habits and insulin sensitivity state that may pose an increased risk of liver disease onset and progression. However, future randomized controlled trials that specifically address potential confounders and employ rigorous study designs can provide more robust evidence regarding the association between adherence to MD and NAFLD.

## Figures and Tables

**Table 1:** Sex, age, lifestyle habits, anthropometric measurements, metabolic parameters and cardio-metabolic indices of the entire study population.

Parameters	N = 336 Mean ± SD or N (%)
**Sex**	
*Male*	126 (37.5)
*Female*	210 (62.5)
**Age (years)**	35.87 ± 10.37
**Lifestyle habits**	
Physical activity	
*Yes*	171 (50.9)
*No*	165 (49.1)
Smoking	
*Yes*	171 (50.9)
*No*	165 (49.1)
**Anthropometric measurements**	
BMI (kg/m^2^)	31.18 ± 9.66
*Normal weight*	145 (43.2)
*Over-weight*	45 (13.4)
*Obesity I*	38 (11.3)
*Obesity II*	40 (11.9)
*Obesity III*	68 (20.2)
WC (cm)	101.81 ± 22.79
* <cut off	149 (44.3)
* >cut off	187 (55.7)
**Metabolic parameters**	
Fasting plasma glucose (mg/dL)	91.79 ± 20.45
Fasting plasma insulin (μU/mL)	11.12 ± 13.65
Total cholesterol (mg/dL)	173.11 ± 41.54
LDL cholesterol (mg/dL)	97.46 ± 43.05
HDL cholesterol (mg/dL)	52.56 ± 13.02
TG (mg/dL)	117.24 ± 51.02
AST (U/L)	25.88 ± 14.03
ALT (U/L)	28.68 ± 20.57
γGT (U/L)	30.38 ± 20.91
**Cardio-metabolic indices**	
HoMA-IR	2.84 ± 3.85
*>2.5*	222 (66.1)
*<2.5*	114 (33.9)
VAI	1.98 ± 1.62
** <cut off	241 (71.7)
** >cut off	95 (28.3)
FLI	53.77 ± 35.43
*<60*	183 (54.5)
*≥* *60*	153 (45.6)

Data are expressed as means ± standard deviation or number (%). BMI, Body Mass Index; WC, Waist Circumference; LDL, Low-Density Lipoprotein; HDL, High-Density Lipoprotein; TG, Triglycerides; AST, Aspartate Aminotransferase; ALT, Alanine Transaminase; γGT; γ-Glutamyltransferase; HoMA-IR, Homeostasis Model Assessment Insulin Resistance; VAI, Visceral Adiposity Index; FLI; Fatty Liver Index; SD, Standard Deviation. * gender specific. ** age specific.

**Table 2:** Response frequency of dietary components included in PREDIMED questionnaire and adherence to MD of the entire study population.

Questions PREDIMED Questionnaire N = 336	n	%
Use of extra-virgin olive oil as main culinary lipid	280	83.3
Extra virgin olive oil > 4 tablespoons	197	58.6
Vegetables ≥ 2 servings/day	171	50.9
Fruits ≥ 3 servings/day	174	51.8
Red/processed meats < 1/day	192	57.1
Butter, cream, margarine < 1/day	153	45.5
Soda drinks < 1/day	186	55.4
Wine glasses ≥ 7/week	155	46.1
Legumes ≥ 3/week	181	53.9
Fish/seafood ≥ 3/week	180	53.6
Commercial sweets and confectionery ≤ 2/week	188	56
Tree nuts ≥ 3/week	152	45.2
Poultry more than red meats	188	56.0
Use of sofrito sauce ≥ 2/week	165	49.1
**PREDIMED categories**		
Low adherence to MD	76	22.6
Average adherence to MD	152	45.2
High adherence to MD	108	32.2

PREDIMED, PREvención con DIetaMEDiterránea; MD, Mediterranean Diet.

**Table 3:** Anthropometric measurements, metabolic parameters and cardio-metabolic indices according to the adherence to MD.

Parameters (N = 336)	Low Adherence to MD (n = 76)	Average Adherence to MD (n = 152)	High Adherence to MD (n = 108)	*p*-Value
Age (years)	36.11 ± 8.81	36.42 ± 10.63	34.93 ± 11.01	0.507
**Anthropometric measurements**				
BMI (kg/m^2^)	39.60 ± 10.98 ^a,b^	31.47 ± 8.19 ^b^	24.86 ± 4.67	**<0.001**
WC (cm)	120.91 ± 26.18 ^a,b^	101.01 ± 20.35 ^b^	89.51 ± 12.22	**<0.001**
* <cut off	15 (19.7%)	66 (43.4%)	68 (63.0%)	χ^2^ = 33.87, ***p* < 0.001**
* >cut off	61 (80.3%)	86 (56.6%)	40 (37.0%)	
**Metabolic parameters**				
Fasting plasma glucose (mg/dL)	104.00 ± 21.50 ^a,b^	91.42 ± 21.10	83.73 ± 13.60	**<0.001**
Fasting plasma insulin (μU/mL)	19.14 ± 15.57 ^a,b^	11.77 ± 14.63 ^b^	4.58 ± 4.70	**<0.001**
Total cholesterol (mg/dL)	194.26 ± 44.28 ^a,b^	175.79 ± 40.09 ^b^	154.44 ± 32.89	**<0.001**
LDL cholesterol (mg/dL)	122.20 ± 46.39 ^a,b^	99.91 ± 39.46 ^b^	76.81 ± 35.10	**<0.001**
HDL cholesterol (mg/dL)	44.64 ± 14.48 ^a,b^	52.68 ± 11.67 ^b^	57.95 ± 13.02	**<0.001**
TG (mg/dL)	146.32 ± 66.77 ^a,b^	116.15 ± 44.74	98.30 ± 35.16	**<0.001**
AST (U/L)	43.02 ± 17.29 ^a,b^	25.04 ± 13.03	21.33 ± 9.90	**<0.001**
ALT (U/L)	36.67 ± 21.24 ^a,b^	26.58 ± 12.26	24.62 ± 26.52	**<0.001**
γGT (U/L)	40.03 ± 24.87 ^a,b^	29.23 ± 17.93	25.20 ± 19.67	**<0.001**
**Cardio-metabolic indices**				
HoMA-IR	5.40 ± 5.09 ^a,b^	2.87 ± 3.67 ^b^	1.00 ± 1.06	**<0.001**
*>2.5*	29 (38.2%)	99 (65.1%)	94 (87.0%)	χ^2^ = 47.65, ***p* < 0.001**
*<2.5*	47 (61.8%)	53 (34.9%)	14 (13.0%)	
VAI	3.11 ± 2.52 ^a,b^	1.84 ± 1.15	1.37 ± 0.76	<0.001
* <cut off	36 (47.4%)	109 (71.7%)	96 (88.9%)	χ^2^ = 37.92, ***p* < 0.001**
** >cut off	40 (52.6%)	43 (28.3%)	12 (11.1%)	
FLI	79.56 ± 30.44 ^a,b^	56.97 ± 34.36 ^b^	31.10 ± 24.67	**<0.001**
<cut off	15 (19.7%)	75 (49.3%)	93 (86.1%)	χ^2^ = 82.18, ***p* < 0.001**
>cut off	61 (80.3%)	77 (50.7%)	15 (13.9%)	

Data are expressed as mean ± standard deviation or number (%). ^a^ vs. intermediate and ^b^ vs. high adherence to MD. A *p*-value in bold type denotes a significant difference (*p* < 0.05). MD, Mediterranean Diet; BMI, Body Mass Index; WC, Waist Circumference; LDL, Low-Density Lipoprotein; HDL, High-Density Lipoprotein; TG, Triglycerides; AST, Aspartate Aminotransferase; ALT, Alanine Transaminase; γGT; γ-Glutamyltransferase; HoMA-IR, Homeostasis Model Assessment Insulin Resistance; VAI, Visceral Adiposity Index; FLI; Fatty Liver Index. * gender specific. ** age specific.

**Table 4:** Anthropometric measurements, metabolic parameters, cardio-metabolic indices and PREDIMED score of the entire study population above and below the cut off of FLI.

Parameters	FLI		*p*-Value
	<60 (n = 183)	≥60 (n = 153)	
Age (years)	33.70 ± 8.44	38.46 ± 11.80	**<0.001**
**Anthropometric measurements**			
BMI (kg/m^2^)	23.99 ± 2.86	39.78 ± 7.68	**<0.001**
WC (cm)	85.73 ± 9.83	121.05 ± 18.59	**<0.001**
* <cut off	132 (72.1%)	17 (11.1%)	χ^2^ = 123.26, ***p* < 0.001**
* >cut off	51 (27.9%)	136 (88.9%)	
**Metabolic parameters**			
Fasting plasma glucose (mg/dL)	82.78 ± 12.14	102.58 ± 23.02	**<0.00** **1**
Fasting plasma insulin (μU/mL)	4.01 ± 9.59	19.64 ± 12.91	**<0.001**
Total cholesterol (mg/dL)	151.56 ± 29.79	198.88 ± 38.90	**<0.001**
LDL cholesterol (mg/dL)	74.03 ± 30.85	125.67 ± 38.55	**<0.001**
HDL cholesterol (mg/dL)	58.84 ± 9.19	45.04 ± 12.97	**<0.001**
TG (mg/dL)	93.48 ± 26.89	145.65 ± 58.13	**<0.001**
AST (U/L)	20.36 ± 6.36	32.48 ± 17.46	**<0.001**
ALT (U/L)	21.68 ± 7.00	37.06 ± 27.29	**<0.001**
γGT (U/L)	23.10 ± 7.77	39.08 ± 27.41	**<0.001**
**Cardio-metabolic indices**			
HoMA-IR	0.85 ± 2.10	5.22 ± 4.11	**<0.001**
<cut off	176 (96.2%)	46 (30.1%)	χ^2^ = 159.52, ***p* < 0.001**
>cut off	7 (3.8%)	107 (69.9%)	
VAI	1.22 ± 0.56	2.89 ± 1.97	**<0.001**
** <cut off	175 (95.6%)	66 (43.1%)	χ^2^=110.64, ***p* < 0.001**
** >cut off	8 (4.4%)	87 (56.9%)	
FLI	23.69 ± 14.00	89.74 ± 11.83	**<0.001**
**PREDIMED score**	9.97 ± 2.33	6.01 ± 2.44	**<0.001**
Low adherence to MD (n, %)	15 (8.2%)	61 (39.9%)	χ^2^ = 45.97, ***p* < 0.001**
Average adherence to MD (n, %)	75 (41.0%)	77 (50.3%)	χ^2^ = 2.57, *p* = 0.109
High adherence to MD (n, %)	93 (50.8%)	15 (9.8%)	χ^2^ = 62.41, ***p* < 0.001**

Data are expressed as mean ± standard deviation or number (%). A *p*-value in bold type denotes a significant difference (*p* < 0.05). BMI, Body Mass Index; WC, Waist Circumference; LDL, Low-Density Lipoprotein; HDL, High-Density Lipoprotein; TG, Triglycerides; AST, Aspartate Aminotransferase; ALT, Alanine Transaminase; γGT; γ-Glutamyltransferase; HoMA-IR, Homeostasis Model Assessment Insulin Resistance; VAI, Visceral Adiposity Index; FLI; Fatty Liver Index; PREDIMED, PREvención con DIetaMEDiterránea. * gender specific. ** age specific.

**Table 5:** Correlations of FLI with anthropometric measurements, metabolic parameters, cardio-metabolic indices and PREDIMED score.

Parameters	FLI (n = 336)			
	Simple Correlation		After Adjusted for HoMA-IR and VAI	
	*r*	*p*-Value	*r*	*p*-Value
Age (years)	0.214	**<0.001**	0.208	**<0.001**
**Anthropometric measurements**				
BMI (kg/m^2^)	0.886	**<0.001**	0.796	**<0.001**
WC (cm)	0.879	**<0.001**	0.740	**<0.001**
**Metabolic parameters**				
Fasting plasma glucose (mg/dL)	0.553	**<0.001**	0.221	**<0.001**
Fasting plasma insulin (μU/mL)	0.641	**<0.001**	0.169	**0.002**
Total cholesterol (mg/dL)	0.644	**<0.001**	0.474	**<0.001**
LDL cholesterol (mg/dL)	0.675	**<0.001**	0.500	**<0.001**
HDL cholesterol (mg/dL)	−0.616	**<0.001**	−0.279	**<0.001**
TG (mg/dL)	0.606	**<0.001**	0.204	**<0.001**
AST (U/L)	0.487	**<0.001**	0.213	**<0.001**
ALT (U/L)	0.414	**<0.001**	0.176	**0.001**
γGT (U/L)	0.427	**<0.001**	0.165	**0.002**
**Cardio-metabolic indices**				
HoMA-IR	0.636	**<0.001**	-	-
VAI	0.583	**<0.001**	-	-
**PREDIMED score**	−0.561	**<0.001**	−0.325	**<0.001**

A *p*-value in bold type denotes a significant difference (*p* < 0.05). BMI, Body Mass Index; WC, Waist Circumference; LDL, Low-Density Lipoprotein; HDL, High-density Lipoprotein; TG, Triglycerides; AST, Aspartate Aminotransferase; ALT, Alanine Transaminase; γGT; γ-Glutamyltransferase; HoMA-IR, Homeostasis Model Assessment Insulin Resistance; VAI, Visceral Adiposity Index; FLI; Fatty Liver Index. PREDIMED, PREvención con DIetaMEDiterránea.

**Table 6:** Multiple regression analysis model (stepwise method) with the PREDIMED score as dependent variable to estimate the predictive value of FLI, VAI and HoMA-IR.

Parameters		Multiple Regression Analysis			
	R	*R* ^2^	*β*	*t*	*p*-Value
**Model 1**					
FLI	0.561	0.313	−0.561	−12.40	**<0.001**
**Model 2**					
FLI	0.561	0.313	−0.416	−7.25	**<0.001**
HoMA-IR	0.589	0.343	−0.229	−3.99	**<0.001**
*Variable excluded: VAI*					
**Model 3**					
FLI categories	0.527	0.276	−0.527	−11.34	**<0.001**
HoMA-IR categories	0.541	0.288	−0.166	−2.59	**0.010**
*Variable excluded: VAI categories*					

A *p*-value in bold type denotes a significant difference (*p* < 0.05). PREDIMED, PREvención con DIetaMEDiterránea.; FLI, Fatty Liver Index; VAI, Visceral Adiposity Index; HoMA-IR, Homeostasis Model Assessment Insulin Resistance.

## Data Availability

The data presented in this study are available on request from the corresponding author. The data are not publicly available due to ethical restrictions.
